# Ultrasound, Dacryocystorhinography and Morphological Examination of Normal Eye and Lacrimal Apparatus of the Donkey (*Equus asinus*)

**DOI:** 10.3390/ani12020132

**Published:** 2022-01-06

**Authors:** Ahmed Abdelbaset-Ismail, Mohamed Aref, Shimaa Ezzeldein, Eslam Eisa, Mudasir Bashir Gugjoo, Ahmed Abdelaal, Hassan Emam, Khalid Al Syaad, Ahmed Ezzat Ahmed, Ali Alshati, Mustafa Abd El Raouf

**Affiliations:** 1 Department of Surgery, Anesthesiology and Radiology, Faculty of Veterinary Medicine, Zagazig University, Zagazig 44519, Egypt; ahmed.abdelbasit.ismail@gmail.com (A.A.-I.); shimaa.ezzeldein@yahoo.com (S.E.); efessa785@gmail.com (E.E.); 2 Department of Anatomy and Embryology, Faculty of Veterinary Medicine, Zagazig University, Zagazig 44519, Egypt; abdelazizanatomy11@gmail.com (M.A.); hassan3anatomy@gmail.com (H.E.); 3 Division of Veterinary Clinical Complex, Faculty of Veterinary Sciences & Animal Husbandry, Sher-e-Kashmir University of Agricultural Sciences and Technology, Jammu & Kashmir, Srinagar 190006, India; mbgugjoo@gmail.com; 4 Department of Animal Medicine, Faculty of Veterinary Medicine, Zagazig University, Zagazig 44519, Egypt; abdelaal79@yahoo.com; 5 Biology Department, Faculty of Science, King Khalid University, P.O. Box 9004, Abha 61413, Saudi Arabia; alsyaad@kku.edu.sa; 6 Director of the Research Center, Faculty of Science, King Khalid University, P.O. Box 9004, Abha 61413, Saudi Arabia; 7 Biology Department, College of Science, King Khalid University, Abha 61413, Saudi Arabia; Aabdelrahman@kku.edu.sa (A.E.A.); Aaalshati@kku.edu.sa (A.A.); 8 Theriogenology Department, Faculty of Veterinary Medicine, South Valley University, Qena 83523, Egypt

**Keywords:** eye, donkey, ultrasonography, dacryocystorhinography, lacrimal gland, nasolacrimal duct

## Abstract

**Simple Summary:**

The role of the eye with lacrimal apparatus as a vital organ of the donkey’s body still needs to be emphasized. The lacrimal gland (LG) is anatomically located at the dorsolateral aspect of the orbit within the lacrimal fossa, and responsible for secretion of the aqueous portion of precorneal tear film. Tears are drained to the exterior through the nasolacrimal duct (NLD), which starts from two puncta lacrimalis at the medial canthus and opens into nasolacrimal sac, and ends by the nasolacrimal orifice (NLO) at the nostril of both sides. Congenital or acquired obstruction of this duct frequently occurs in horses and leads to epiphora. Therefore, the aim of the present study was to investigate the normal macromorphological and ultrasonographic features of the eye and lacrimal gland, as well as normal dacryocystorhinography of the donkey (*Equus asinus*) in Egypt. The results illustrated the ultrasonographic biometric values of the eye, as well as the shape and size of the lacrimal gland. In addition, dacryocystorhinography and normal dissecting anatomy of the lacrimal apparatus was illustrated. The results of this study may act as the baseline for proper management of conditions of the eye and lacrimal apparatus in the donkey in the future.

**Abstract:**

The study investigated normal macromorphological and ultrasonographic features of the eye and lacrimal gland, as well as normal dacryocystorhinography of the donkey (*Equus asinus*) in Egypt. A total of 36 donkeys of different ages, weights, and sexes were included in the study: 21 live animals for ultrasonography and dacryocystorhinography, and 15 cadaver skulls for morphological anatomy of the lacrimal apparatus. The ultrasound biometric values of the eye were 33.7 ± 1.7 mm for axial globe length (AGL), 39.8 ± 2.1 mm for globe diameter (GD), 10.8 ± 0.7 mm for lens thickness (LT), 3.2 ± 0.7 mm for anterior chamber depth (ACD), and 19.3 ± 1.6 mm for vitreous chamber depth (VCD). The lacrimal gland was recognized as a hypoechogenic structure with an anechoic core, located at the dorsolateral aspect of the orbit, and ovoid in shape. The mean NLD length was 193.0 ± 9.8 mm by radiography and 206.0 ± 20.4 mm by gross assessment. One NL orifice (NLO) was noticed on each side, with a diameter of 3.0 ± 0.1 mm and located 12.1 ± 2.1 mm from the dorsal commissure of the nostril. These results may act as the baseline for proper management of conditions of the eye and lacrimal apparatus in the donkey in the future.

## 1. Introduction

Unlike the horse, there is a paucity of published scientific reports on another member of the Equidae family, the donkey. The donkey is different from the horse in some details of anatomic conformation. Thus, there is a need to expand our understanding about the donkey, in particular its anatomical structures and their proper interpretation using various diagnostic tools. The most beneficial, inexpensive, and available tools that strongly aid in diagnosis of various soft-tissue-derived disorders are ultrasonography and contrast radiography [1,2]. Additionally, the unavailability and high cost of magnetic resonance imaging (MRI) and computed tomography (CT) currently make the US and radiography the only applicable tools in the large animals, including donkeys [3,4].

To expand knowledge of a lesser-studied species, the normal soft tissue architecture and dimensions using either ultrasonography or contrast radiography are definitely required for proper diagnosis and prognosis of soft tissue disorders. Ultrasonographic findings and measurements have been described in the donkey for normal pregnancy, synovial-containing structures in normal and illness cases, cardiovascular disease, total splenectomy, and for measuring adipose tissue reserves [5,6,7,8,9].

The eye and associated lacrimal apparatus as a vital organ of the donkey’s body still needs much research emphasis. In horses, the eyeball (or globe) consists of three layers: outer fibrous, middle, and internal. The outer fibrous layer consists of sclera (white of the eye) and cornea (transparent part of the eye). The middle layer (uvea) consists of the iris (colored part), choroid (vascular portion), and ciliary body (the part responsible for the secretion of aqueous humor, and which controls the lens thickness). The internal layer (nervous layer) consists of the retina with photoreceptors; their nerve fibers are bundled together to form the optic nerve. Other intraocular structures are the lens, as well as the aqueous- and vitreous-containing chambers [10,11].

The LG is anatomically located at dorsolateral aspect of orbit within the lacrimal fossa, and is responsible for secretion of aqueous portion of the precorneal tear film [12,13,14]. Tears are drained to the exterior through the NLD, which starts from two puncta lacrimalis at the medial canthus and opens into nasolacrimal sac, and ends at the NLO within each nostril [15]. Congenital or acquired obstruction of this duct frequently occurs in horses and leads to a symptom known as epiphora [16]. Ultrasonographic studies reporting abscessation of the lacrimal gland or dacryoadenitis have been reported in horses [17,18]; however, no data so far has been reported in the donkey.

To the best of our knowledge, so far only two reports showing the normal eye biometry of the donkey, as well as the variability of the biometrics as per age and weight dependence, have been reported [19,20]. However, the morphometric data of LG ultrasound and dacryocystography of the donkey were beyond their scope. In horses, ultrasound has been used for diagnosis and characterization of various intraocular diseases (e.g., lens displacement, cataract, floaters, etc.) [2,21,22]. For this reason, the current study was performed to show the normal macromorphological and ultrasonographic appearance of the eye and LG, as well as normal dacryocystorhinography of the donkey (*Equus asinus*) in Egypt.

## 2. Materials and Methods

### 2.1. Ethical Approval

The current work was performed in accordance with the rules of ethical committee and animal welfare of the Faculty of Veterinary Medicine, Zagazig University, Egypt.

### 2.2. Animals

Apparently healthy donkeys (*n* = 36) of both sexes and aged between 3 to 5 years (mean ± standard deviation: 4.2 ± 0.8 years) were the subject of this study. A total of 21 clinically healthy live animals were used for ultrasonographic and radiographic examinations, while 15 animals were used as cadavers for anatomical studies. All the imaging procedures were performed at the Imaging Unit, Department of Surgery, Anesthesiology, and Radiology, Faculty of Veterinary Medicine, Zagazig University; and were conducted according to the institutional guidelines for regulation of the care and use of animals. The animals were sedated intravenously (I/V) using xylazine hydrochloride 2% (Xylaject; ADWIA CO, 10th of Ramadan, Egypt) at a dose of 1 mg/kg b.wt. in all approaches except for the ocular ultrasonography.

### 2.3. Clinical Examination

Each animal was initially subjected to thorough clinical examination of both eyes for any abnormalities. The details of the location of the nasal punctum and lacrimal puncta were recorded.

### 2.4. Ultrasonographic Examination

Transpalpebral and transcorneal ultrasonographic scanning of the right and left eyes of each animal were performed using an ultrasonographic machine (SonoScape A5V ultrasound machine, Guangdong, China) connected to a linear transducer and at a 7 MHz frequency. These approaches were done under the effect of both topical ocular anesthesia and auriculopalpebral nerve block. The different structures of the globe were scanned for their ultrasonographic appearance and dimensions. In addition, the location, appearance, and dimensions of the LG were also determined.

### 2.5. Radiographic Examination (Dacryocystorhinography)

Contrast radiography using iodinated contrast media (Telebrex^®^, Amoun Pharmaceutical Co., Cairo, Egypt) was performed for each NLD to determine its pathway and dimensions. The contrast medium was retrogradely infused into the NLD using a 3.5 French, open-end, tomcat catheter that was 3 inches in length at an amount of 2.5 ± 0.5 mL/animal. Immediately after infusion, lateral radiographic imaging of the skull was performed using an average of 6.3 mAs and an average of 70 kVp.

### 2.6. Anatomical Studies

For topographical examination, fresh dissection of the head (*n* = 15) was done to study the following points: (i) location, shape, and color of the LG, accessed by dissecting the upper eyelid skin off from deeper tissues, then removing the supraorbital process and peri-orbital fascia; and (ii) gross morphology of the NL apparatus’ particularly normal anatomical pathway, relations, and position of NLD, which was achieved after removal of the lateral wall of the maxillary sinus. For morphometric analysis, the dimensions of the LG; the length of the caudal, middle, and rostral part of the NLD; the diameter of the NL orifice, and the distance of the NL orifice from the dorsal nasal angle were done in all animals using a stainless caliper and a ruler. Next, a descriptive statistical analysis was preformed using SPSS version 25 (SPSS IBM Corp, Armonk, NY, USA) to obtain the mean ± SD of all obtained measurements. In all images, the structures were described, interpreted, and then labeled based on the *Nomina Anatomica Veterinaria* [23]. For determining the pathway of the NLD, bone preparation of one fresh head was carried out by the maceration method, as shown in previous studies [24].

## 3. Results

### 3.1. Ultrasonographic Findings

**Figure 1:**
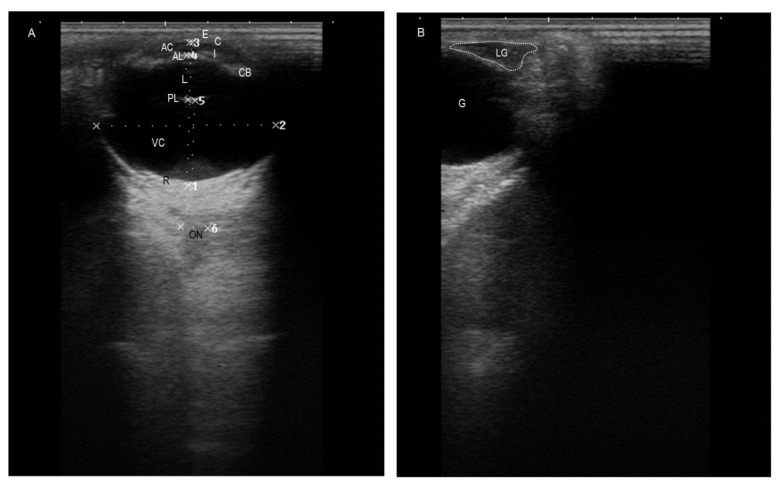
(**A**) A representative ultrasonogram depicting normal eye structures and their measurements in a healthy adult donkey. (**B**) A representative ultrasonogram demonstrating shape and location of the normal LG of a healthy adult donkey. E: eyelid; C: cornea; AC: anterior chamber; AL: anterior lens; L: lens; PL: posterior lens; I: iris; CB: ciliary body; VC: vitreous chamber; R: retina; ON: optic nerve; G: globe; LG: lacrimal gland.

The ultrasonographic biometric analysis of the eye components and LG are shown in Table 1. In addition, Figure 1 depicts the normal appearance of the eye structures (Figure 1A) and the LG (Figure 1B) on ultrasonography. The cornea was clearly visualized via a transpalpebral or transcorneal approach. It appeared as a thin curved structure with homogenous echogenicity. During the examination, much care was taken to avoid compression of the ultrasound signal of the cornea due to pressure from the transducer. The aqueous humor appeared homogenously anechoic, without any evidence of the presence of echogenic materials (fibrin or sepsis). The anterior chamber depth (ACD) had an average depth of 3.2 ± 0.5 mm. The anterior and posterior capsules of the lens appeared echogenic of only one regular line for each. The lenticular core appeared anechoic. The average lens dimension (distance between the anterior and posterior lens capsule at the axis, or widest point) was 10.8 ± 0.7 mm. The iris and ciliary body were seen posterior to the AC and anterior to the lens. Vitreous humor appeared within the vitreous chamber (VC) as a uniform anechoic structure. The average vitreous chamber depth (VCD) was 19.3 ± 1.6 mm. There was no evidence of presence of hypo- to hyperechogenic structures (blood, inflammatory exudate, fibrin, vitreal floaters, or tumorigenic mass). It was difficult to distinguish the retina, choroid, and sclera separately. However, the retina appeared medially of hyperechoic appearance. Regarding retrobulbar structures, the optic nerve, periorbital muscles, and retrobulbar fat pad were clearly visualized. The optic nerve appeared hypoechoic, and its optic disc appeared hyperechoic. The retrobulbar fat pad surrounding the optic nerve appeared heterogenous, with hyperechoic threads in between. The LG was clearly visualized via a transpalpebral approach at the dorsolateral portion of the orbit, underneath the supraorbital process and within the lacrimal fossa. The LG appeared ovoid, with a hypoechoic structure and anechoic core. Measurements of the length of the gland in the anteroposterior direction and the diameter of the gland in the mediolateral direction were performed. The mean length of the LG was 16.9 ± 1.6 mm, with a maximum of 20.1 mm and a minimum of 14.3 mm; the mean diameter of the LG was 6.1 ± 0.3 mm, with a maximum of 6.4 mm and a minimum of 5.1 mm.

### 3.2. Dacryocystorhinography

**Figure 2:**
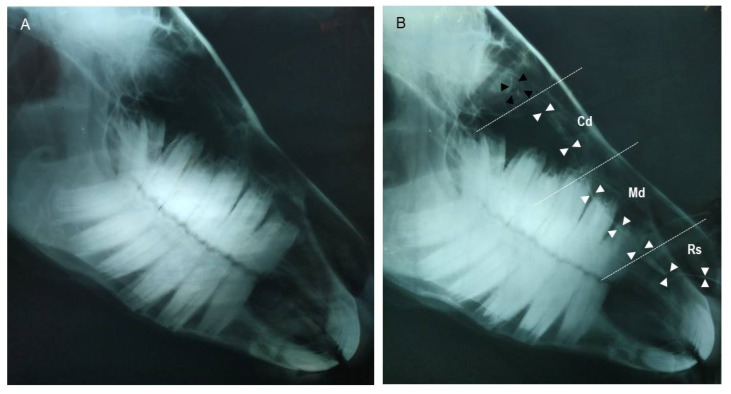
(**A**) Representative lateral plain radiograph of the head region of a healthy adult donkey. (**B**) Lateral contrast radiograph of nasolacrimal drainage system (dacryocystorhinography) outlining that the NL duct (whit arrow heads) has 3 portions according to the width change during its course, as well as the NL sac (black arrow heads) of a healthy adult donkey. Rs: rostral cartilaginous segment; Md: middle membranous segment; Cd: caudal osseous segment.

Since it was harder to detect the NLD by plain radiography (Figure 2A), the contrast radiography (dacryocystorhinography) was performed in order to clearly visualize the NLD and NL sac (Figure 2B). On the radiograph, the course of the NLD appeared tortuous. The caudal osseous segment continued distally from the NL sac approximately at the bases of the upper 3rd–1st molars, then narrowed gradually until reaching the base of the upper 2nd premolar (middle membranous segment), then dilated at its rostral segment at the base of the 1st premolar tooth (rostral cartilage portion), which curved laterally over the incisive bone process to open at the NLO. The upper and lower 95% confidence interval (CI) and the average readouts of the whole NLD length, as well as dimensions of the rostral cartilaginous, middle membranous, and caudal osseous segments of the NLD, are shown in Table 1. As shown, we found the rostral cartilaginous segment was the widest (6.7 ± 0.5 mm in diameter), whereas the caudal segment was the narrowest (6.7 ± 0.5 mm in diameter) (Table 1 and Figure 2B). The NL sac appeared in the dacryocystography as a small round (slight dilation) sac within the osseous fossa formed by the lacrimal and maxillary bones, approximately at the level of root of the upper 3rd molar tooth, as shown in Figure 2B.

### 3.3. Macromorphological Findings for the Lacrimal Apparatus

**Figure 3:**
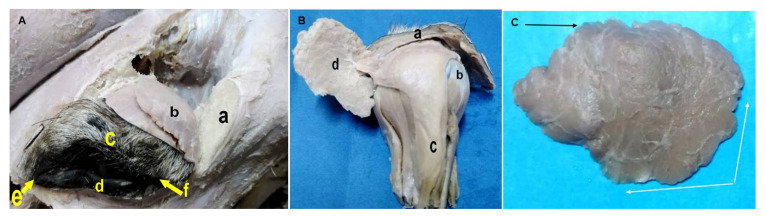
(**A**) A photomacrograph of a dissected formalin-fixed donkey head (after breaking the supraorbital process of the frontal bone), showing the broken supraorbital process of the frontal bone (a), lacrimal gland (b), upper eyelid (c), lower eyelid (d), medial canthus (e), and lateral canthus (f). (**B**) A photomacrograph of a dissected formalin-fixed donkey eye after removal from the orbit showing the upper eyelid (a), eyeball (b), extraocular muscles (c), and lacrimal gland (reflected, d). (**C**) A photomacrograph of an isolated formalin-fixed lacrimal gland of the donkey showing cranial regular border (white arrows) and caudal serrated border (black arrow).

The lacrimal apparatus in the donkey consisted of secretory and excretory divisions. The secretory one included the LG and excretory ductules. The excretory division included the lacrimal caruncle, lacrimal puncta, lacrimal canaliculi, lacrimal sac, and NLD and NLO. As shown in Figure 3, the LG was found at the dorsolateral portion of the globe inside a fossa underneath the supraorbital process of the frontal bone, and was incompletely covered with periorbital adipose tissue. It was ovoid, lobulated, and flattened, with irregular borders. It also variably appeared from whitish yellow to light brown in color. It had two borders: the rostral border (located under the rim of the upper eyelid, regular, and flattened) and caudal border (serrated). In addition, it had two surfaces: the dorsal convex and ventral concave surfaces.

**Figure 4:**
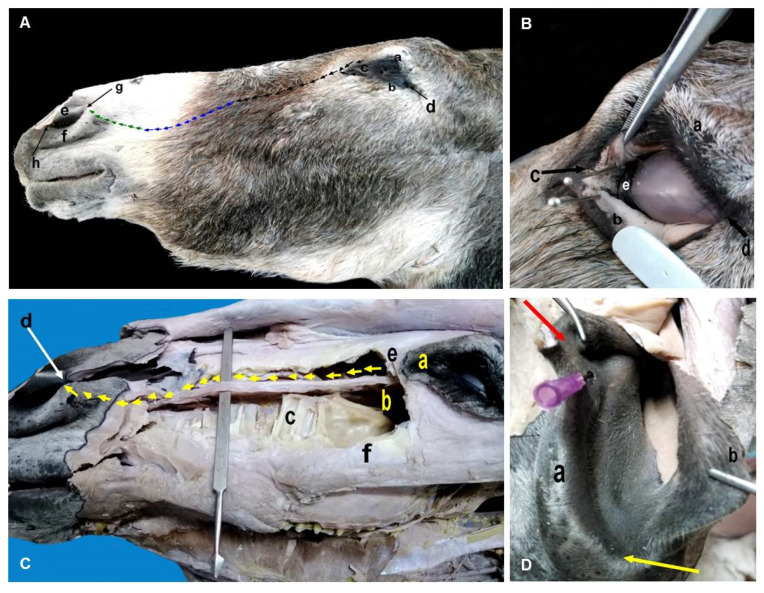
(**A**) A photomacrograph of a formalin-fixed donkey head showing the upper eyelid (a), lower eyelid (b), medial canthus (c), lateral canthus (d), medial nasal alea (e), lateral nasal alea (f), dorsal nasal commissure (g), ventral nasal commissure (h), pathway of nasolacrimal duct inside caudal osseous canal (black arrows), middle membranous part (blue arrows), and rostral terminal cartilaginous part (green arrows). (**B**) A photomacrograph of a formalin-fixed donkey head showing the upper eyelid (a), lower eyelid (everted) (b), medial canthus (c), lateral canthus (d), third eyelid (e), and two lacrimal puncta (needles inserted). (**C**) A photomacrograph of a formalin-fixed donkey head after removal of the maxilla bone showing the medial canthus (a), maxillary sinus containing infraorbital canal (b), roots of upper molar teeth (c), dorsal nasal commissure (d), lacrimal bone (e), maxilla bone (f), and nasolacrimal duct (demarcated with yellow arrows). (**D**): A photomacrograph of a formalin-fixed donkey nostril after opening the dorsal nasal commissure, showing the medial nasal alea (a), lateral nasal alea (b), dorsal nasal commissure (red arrow), ventral nasal commissure (yellow arrow), and nasolacrimal orifice (inserted needle).

Table 2 shows the weight and dimensions of the LG in the donkey, with minimum and maximum values for each parameter. The LG possessed 8 to 10 excretory ducts that originated from its ventral aspect, then opened at the dorsal conjunctival fornix. Concerning the excretory division (Figure 4A), there were inferior and superior oval orifices per each eye, namely the lacrimal puncta, close to the lacrimal caruncle at the medial canthus. These puncta connected with their respective lacrimal canaliculi over the conjunctival surface of the upper and lower eyelids, and then opened into a structure called the lacrimal sac (Figure 4B). The lacrimal sac was funnel-like, and was located in the bony fossa of the lacrimal bone, then continued as the NLD.

The NLD originated at the base of the lacrimal sac and had three segments: the caudal “osseous”, middle “membranous”, and rostral “cartilaginous and terminal” segments. Figure 4C,D demonstrate the course of the NLD in the lacrimal groove and its relation to the surrounding structures until dorsally curved in the cavernous tissue and plate of cartilage of the nasal fold, to externally open on the lateral wing of the nasal vestibule via the NLO. As shown in Table 2, the mean length of the NLD was 206.0 ± 20.4 mm in the anterio-posterior direction. The mean lengths of the caudal osseous, middle membranous, and rostral cartilaginous segments were 74.8 ± 8.6 mm, 45.3 ± 6.9 mm, and 85.2 ± 8.2 mm, respectively. There was only one NLO per each nostril on the medial wall of the nasal vestibule, at the muco-cutaneous junction by the nasal orifice (Figure 4D). The mean diameter of the NLO was 3.9 ± 0.9 mm, and the mean distance from the dorsal commissure of the nostril to the NLO was 12.1 ± 2.2 mm (Table 2).

## 4. Discussion

The most important findings of this study were that the lacrimal gland and nasolacrimal duct, for the first time, were readily shown and measured by the aid of transpalpebral ultrasonography and contrast radiography, respectively. This study also provided biometric measurements of the normal eye of an Egyptian donkey with the aid of ultrasonography.

The ocular biometric values have been reported in different animals, including horses [25,26], cattle [27], camels [28,29], sheep [30], goats [31], dogs [32,33], cats [34,35], Asian elephants [36], cinereous vultures [37], and the striped owl [38]. However, to the best of the authors’ knowledge, there are only two peer–reviewed reports that have been done by one research team that present biometric values of a normal donkey’s eye [19,20]. However, the data on extraocular structures, particularly the lacrimal gland and nasolacrimal drainage system, have not been explained yet.

This study was designed using donkeys because: (i) there is no rich scientific work for them in comparison to other animal species; (ii) they are different from horses in some of their anatomical conformations; and (iii) as a draft animal, they play a potential role in developing communities for both agriculture and riding purposes. The ultrasound is a diagnostic tool by which the critical data of intervention, follow-up monitoring, and prognosis can be obtained, particularly when other routine ophthalmologic investigations are not sufficient. Furthermore, it can be used in an unanesthetized animal for examination of the intraocular and retrobulbar structures [22].

Ultrasound of the LG has been reported in horses for detection of abscesses or dacryoadenitis [17,18]. For this reason, this study was done to report normal imaging of the LG in the donkey that is missing so far in the scientific literature. Nevertheless, further studies are needed to evaluate whether there are biometric alterations of the LG in relation to gender and age in donkeys, since the gland is directly targeted by sex hormones [39]. Our data showed that the LG appeared ovoid, and the average length was (US: 16.9 ± 1.6 mm; grossly: 25.1 ± 3.9 mm), and diameter (US: 6.1 ± 0.3 mm). The average width of the LG was 34.7 ± 4.9 mm in gross examination; whereas in horses, the LG thickness was 3.2–3.3 mm [40]. In addition, in horses, the LG measures about 5 cm transversely and 2.5–3.0 cm in the sagittal direction [41,42,43]. In this study, the LG appeared ovoid, light brown, and partially lined with adipose tissue, and occupied the dorsolateral quadrant of the globe beneath the supraorbital and zygomatic process of frontal bone [44]. Herein, the LG possessed 8–10 excretory ducts, while in horses, the excretory ducts are 12–16 in number [41].

It has been reported that dacryocystorhinography (contrast radiography of nasolacrimal duct) in horses [15], camels [45], llamas [46], sheep [47], dogs [48,49], and cats [48] was performed; these studies provided proper assessment of the normal radiographic characteristics of the NLD.

The parts of nasolacrimal system include two canaliculi, the nasolacrimal sac, and the NLD. In horses and mules, the lacrimal sac (saccus lacrimalis) locates the funnel-like fossa of the osseous lacrimal duct as a dilating portion of the nasolacrimal duct [41,50].

The NLD runs rostrally beneath the zygomatic and maxillary bone inside the osseous lacrimal canal and terminates at the margin of the conchal crest [43]. In horses, the course of the distal NLD is likely to be straight; its ostium is located on the floor of the nasal vestibule at the mucocutaneous junction [42]. Unlike in horses, as shown by this study, the course of the NLD was found to be rostrally labyrinthine (“tortuous”), and opened at the medial wall of the nasal vestibule at the mucocutaneous junction by the nasal orifice (about 3.9 ± 0.9 mm in diameter) of the nasolacrimal duct. Another study reported that the diameter of nasal orifice of NLD was 3 mm [44].

In equids, anatomy of the NL drainage system must be taken into consideration before carrying out any common surgeries in the head region, such as treatment of nasal lacerations and redundant alar folds, or sinus trephining [51]. Interestingly, the NLD was also found to be just dilated (4.5 ± 0.2 mm) at the level of the maxillary cheek teeth as detected by contrast radiography. This explained why the full retrograde catheterization of the nasolacrimal duct of the donkey beyond the 2nd–3rd maxillary cheek teeth (2.5 ± 0.6 mm) is problematic. The chronic epiphora experienced in the reported cases of internal obstruction (e.g., atresia, dacryolith) or even external obstruction (e.g., maxillary sinus empyema) in horses implies the potential role of nasolacrimal duct radiography as a diagnostic and prognostic method for both horses and donkeys to evaluate its patency [15]. Difficult visualization of the lucent nasolacrimal duct by plain radiography due to osseous superimposition made contrast radiography convenient and superior. In addition, we found that the rostral part of nasolacrimal duct was wide, measuring 67.4 ± 4.9 mm in length. This suggested that it is only possible to cannulate the NLD in the donkey for retrograde flushing or even injection of contrast medium using catheters or cannulas that should be sorted according to the measurements reported here, particularly the rostral portion of the NLD.

Herein, the NLO was found in the medial wall of the nasal vestibule at the muco-cutaneous junction. Unlike in the donkey, the NLO remains laterally and in the floor of the nostril near the transition to the mucous membrane in the horse [43], and in the internal cutaneous tissue of the lateral wall of the external nares in 87.5% of mules [50]. The length of the NLD was 206.0 ± 20.4 mm in the anterio-posterior direction, whilst in horse, the duct measures approximately 250 to 300 mm in length [41].

## 5. Conclusions

Herein, the normal structural features of the eye and its drainage system of *Equus asinus* in Egypt were obtained using anatomical, ultrasonographic, and radiographic examinations. Thereby, this study added to the literature that will help in proper examinations, and consequently in reaching proper diagnoses and prognoses, of the disorders that could affect eye and its drainage system in the donkey.

## Figures and Tables

**Table 1:** Descriptive statistical analysis of the globe, lacrimal gland, and NLD biometric measurements on ultrasonographic and radiographic imaging of 21 clinically normal live donkeys.

Variable (mm)		95% Confidence Interval (CI)		Mean (Standard Deviation)
		Lower 95% CI of Mean	Upper 95% CI of Mean	
Ultrasonography	Axial globe length (AGL)	30.4	36.1	33.7 (1.7)
	Globe diameter (GD)	35.7	43.1	39.8 (2.1)
	Lens thickness (LT)	10.1	12.5	10.8 (0.7)
	Anterior chamber diameter (ACD)	2.5	3.9	3.2 (0.5)
	Vitreous chamber diameter (VCD)	16.3	21.9	19.3 (1.6)
	Lacrimal gland length (LGL)	14.3	20.1	16.9 (1.6)
	Lacrimal gland diameter (LGD)	5.1	6.4	6.1 (0.3)
Radiography	Nasolacrimal duct rostral (NLDRs) diameter	4.1	4.9	4.5 (0.2)
	Nasolacrimal duct middle (NLDMd) diameter	2.2	2.9	2.6 (0.2)
	Nasolacrimal duct caudal (NLDCd) diameter	0.8	1.5	1.1 (0.2)
	Nasolacrimal duct (NLD) length	172.0	209.0	193.0 (9.8)
	Nasolacrimal duct (NLD) rostral cartilaginous	60.0	79.0	67.4 (4.9)
	Nasolacrimal duct (NLD) middle membranous	60.0	81.5	67.9 (6.0)
	Nasolacrimal duct (NLD) caudal osseous	60.0	79.0	67.2 (5.1)

**Table 2:** Descriptive statistical analysis of gross-derived lacrimal apparatus measurements of clinically normal dead donkeys.

Variable	95% Confidence Interval (CI)		Mean (Standard Deviation)
	Lower 95% CI of Mean	Upper 95% CI of Mean	
Lacrimal gland weight (LGW) (gm)	2.5	3.5	2.9 (0.2)
Lacrimal gland length (LGL) (mm)	19.0	31.5	25.1 (3.9)
Lacrimal gland width (LGD) (mm)	28.0	43.0	34.7 (4.9)
Nasolacrimal duct (NLD) length (mm)	180.0	245.0	206.0 (20.4)
Nasolacrimal duct (NLD) rostral cartilaginous (mm)	75.0	95.0	85.2 (8.2)
Nasolacrimal duct (NLD) middle membranous (mm)	40.0	65.0	45.3 (6.9)
Nasolacrimal duct (NLD) caudal osseous (mm)	60.0	85.0	74.8 (8.6)
Nasolacrimal (NL) orifice diameter (mm)	3.0	5.0	3.9 (0.9)
Distance from dorsal commissure to NL orifice (mm)	9.0	16.0	12.1 (2.2)

## Data Availability

The data presented in this study are available on request from the corresponding author.
